# Spectroscopic properties and molecular structure of copper phytate complexes: IR, Raman, UV–Vis, EPR studies and DFT calculations

**DOI:** 10.1007/s00775-018-1622-0

**Published:** 2018-10-24

**Authors:** A. Zając, L. Dymińska, J. Lorenc, S. M. Kaczmarek, G. Leniec, M. Ptak, J. Hanuza

**Affiliations:** 1 Department of Bioorganic Chemistry, Faculty of Engineering and Economy, Institute of Chemistry and Food Technology, Wroclaw University of Economics, 118/120 Komandorska Str., 53-345 Wrocław, Poland; 2 Institute of Physics, West Pomeranian University of Technology, Al. Piastów 48, 70-310 Szczecin, Poland; 3 Institute of Low Temperature and Structure Research, 2 Okólna Str., 50-422 Wrocław, Poland

**Keywords:** Copper phytates, Infrared and FT-Raman spectroscopy, Electron absorption spectroscopy, DFT quantum chemical calculations, Electron paramagnetic resonance

## Abstract

The copper phytate IP6Cu, IP6Cu_2_ and IP6Cu_3_ complexes were synthesized changing the phytate to metal mole ratio. The obtained products have been characterized by means of chemical and spectroscopic studies. Spectroscopic ATR/IR, FT-Raman, UV–Vis, EPR and magnetic measurements were carried out. The structures of these compounds have been proposed on the basis of the group theory and geometry optimization taking into account the shape and number of the bands corresponding to the stretching and bending vibrations of the phosphate group and metal–oxygen polyhedron. The role of the inter- and intra-hydrogen bonds in stabilization of the structure has been discussed. EPR studies showed that a local rhombic symmetry of copper ions appears in the studied phytates. Dominant interactions show antiferromagnetic properties depending on the content of paramagnetic ions.

**Electronic supplementary material:**

The online version of this article (10.1007/s00775-018-1622-0) contains supplementary material, which is available to authorized users.

## Introduction

Phytic acid and its salts play an important role in human and non-ruminants diets and bioavailability or assimilation of some food components. They are a principal storage form of phosphorus compounds naturally occurring in plants. For example, poultry diet is mainly based on seeds and plant-based materials. Moreover, in human diet legumes form an essential part of the nourishment containing a significant amount of phytic acid, which exists intrinsically as phytate in the anionic form in plants. Phytates appear mainly as a complex salt of metal cations bound to proteins and starch. Copper(II) ions play an important micronutrient role in plant nutrition showing several functional contributions as enzyme activator, major function in photosynthesis and reproductive stage, in respiratory enzymes and chlorophyll production, cause an increase of sugar content, intensify color and improves flavor in fruit and vegetables [1]. In our previous work on silver phytates [2], their environmental, consuming and medicinal importance was reviewed [3–8].

Phytate anions and their complexes affect availability of phosphor and other trace nutrients in plants [9–12]. Phytin complexes usually appear in the germ of corn, the aleurone layer and outer bran of wheat and rice, globoids in legumes and oils from seeds [13–15]. Phytic acid in these natural products inhibits the bioavailability of minerals forming insoluble complexes with the metallic cations, and in consequence impeding the hydrolytic enzymes. Therefore, the necessity of rapid detection and quantification of phytic derivatives in food makes searching for new methods important. The present work proposes an application of several spectroscopic methods for studying copper phytates. It is a continuation of our previous studies on silver phytates [2]. We expect to confirm the conclusions drawn in the work on structure and spectroscopic properties of the phytate complexes. Instead of Ag^+^ ions, the complexes studied in the present work contain Cu^2+^ ions for which the vibrational and electron spectra have been measured. Due to the magnetic properties of copper(II), EPR and magnetic studies have been performed. The obtained spectroscopic data were discussed in terms of quantum chemical DFT calculations. We believe that the results presented here will be useful in identification of different forms of the phytate copper complexes in plants and human-originated materials.

## Experimental

### Materials

**Fig. 1:**
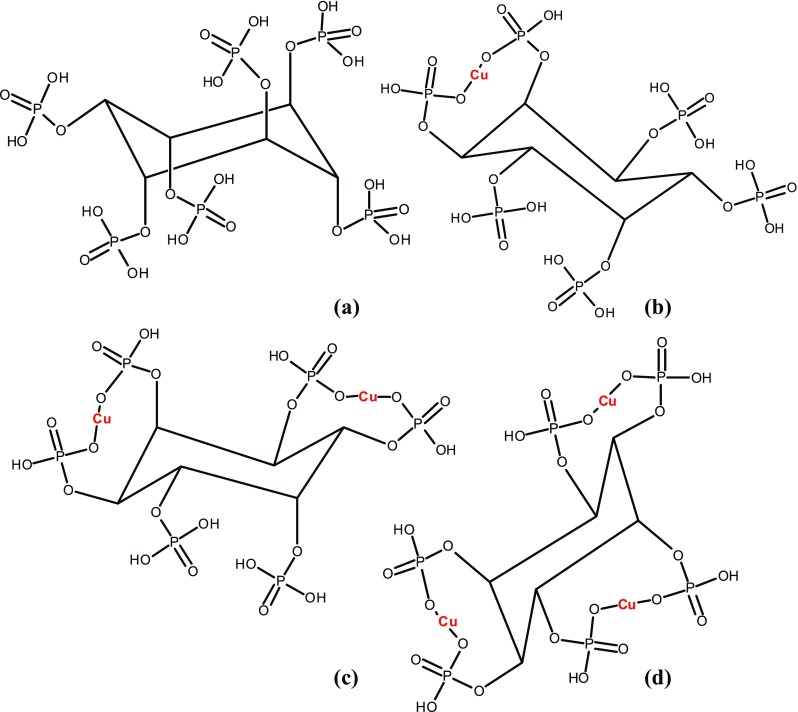
The chemical structure of the IP6 (**a**), IP6Cu (**b**), IP6Cu_2_ (**c**) and IP6Cu_3_ (**d**)

The copper phytate complexes were synthesized changing phytic acid to metal mole ratio. In this method, a sample of 5 ml of aqueous solution of phytic acid (50 wt% solution in water, Sigma-Aldrich cat. no. 593648) was mixed thoroughly with 0.75, 1.5 and 2.26 g copper carbonate (Sigma-Aldrich cat. no. 85150) at room temperature for 2–3 h. After complexation, the content of the beaker was freeze dried. The freeze-drying process was conducted by treating the reaction products at 194 K for 24 h, followed by drying them at the pressure 0.02 mbar for 48 h using a Labconco FreeZone laboratory freeze dryer 4.5 L (the USA). The final products were obtained in the form of resin, the XRD structure determination of which was impossible. Figure 1 presents the chemical structures of the compounds studied in this work, i.e., IP6=C_6_H_6_(PO_4_H_2_)_6_; IP6Cu=Cu[C_6_H_6_(PO_4_H_2_)_4_(PO_4_H)_4_]; IP6Cu_2_=Cu_2_[C_6_H_6_(PO_4_H_2_)_2_(PO_4_H)_4_] and IP6Cu_3_=Cu_3_[C_6_H_6_(PO_4_H)_6_].

### IR and Raman spectra measurements

IR spectra were measured using a Nicolet iS50 FT-IR (Thermo Scientific) spectrometer equipped with an Automated Beamsplitter exchange system (iS50 ABX containing a DLaTGS KBr detector and a DLaTGS Solid Substrate detector for mid-IR and far-IR regions, respectively). Built-in all-reflective diamond ATR module (iS50 ATR), Thermo Scientific Polaris™ and HeNe laser as an IR radiation source. Polycrystalline mid-IR spectra were collected in the 4000–400 cm^−1^ range in KBr pellets and far-IR spectra in the 600–50 cm^−1^ range in Nujol mull. Spectral resolution was set to 4 cm^−1^.

Raman spectra in the 4000–80 cm^−1^ range were measured in back scattering geometry with a FT Bruker 110/S spectrometer. The resolution was 2.0 cm^−1^. The YAG:Nd (excitation wavelength 1064 nm) laser was used as an excitation source.

### Electron absorption spectra

Room temperature electron absorption spectra were measured in the 200–1500 nm spectral range using a Cary-Varian 5E UV–Vis–near-IR spectrophotometer. In the case of a weak signal of the spectrum, the spectrophotometer was switched to measurements in the diffuse reflectance mode. Diffuse reflectance spectra were recorded with a Praying Mantis diffuse reflectance accessories. In these measurements, the base line was first recorded for Al_2_O_3_ powder, and next this line was subtracted from the spectra that had been obtained for the particular powder sample.

### Quantum DFT calculations

The geometry optimization of the molecular structure of the studied compound was performed for a monomeric unit with the use of Gaussian 03 program package [16]. All calculations were performed using density functional three-parameter hybrid (B3LYP) methods [17–19] with the 6-31G(d,p) [20] basis set starting from the X-ray geometry. The calculated and experimental values were compared using scaling factors to correct the evaluated wavenumbers for vibrational anharmonicity and deficiencies inherent to the used computational level. The animations of the molecular vibrations, NBO energies and other quantum chemical data were performed using the ChemCraft program [21, 22].

A linear correlation was used for scaling the theoretical wavenumbers to compare them with the experimental values. 0.96 scaling factor was used for the range 3500–2500 cm^−1^, 0.94 for the range 2499–1000 cm^−1^ and 1.00 below 1000 cm^−1^.

### EPR spectra and magnetic properties

EPR spectra were recorded in the full temperature range 3–300 K using a conventional X-band Bruker ELEXSYS E 500 CW-spectrometer operating at 9.5 GHz with 100 kHz magnetic field modulation. The magnetic field was changed from 0 to 1.4 T. The investigated samples were in a nanocrystallite powder form. The first derivative of the powder absorption spectra was recorded as a function of the applied magnetic field. Temperature dependence of the EPR spectra of the powder sample in the 3–300 K temperature range was recorded using an Oxford Instruments ESP helium-flow cryostat. The SIMPOW6 program was applied to fit spin Hamiltonian parameters [23] and EPR-NMR software to generate the EPR spectrum [24].

## Results and discussion

### Molecular structures of the studied copper phytate complexes

**Fig. 2:**
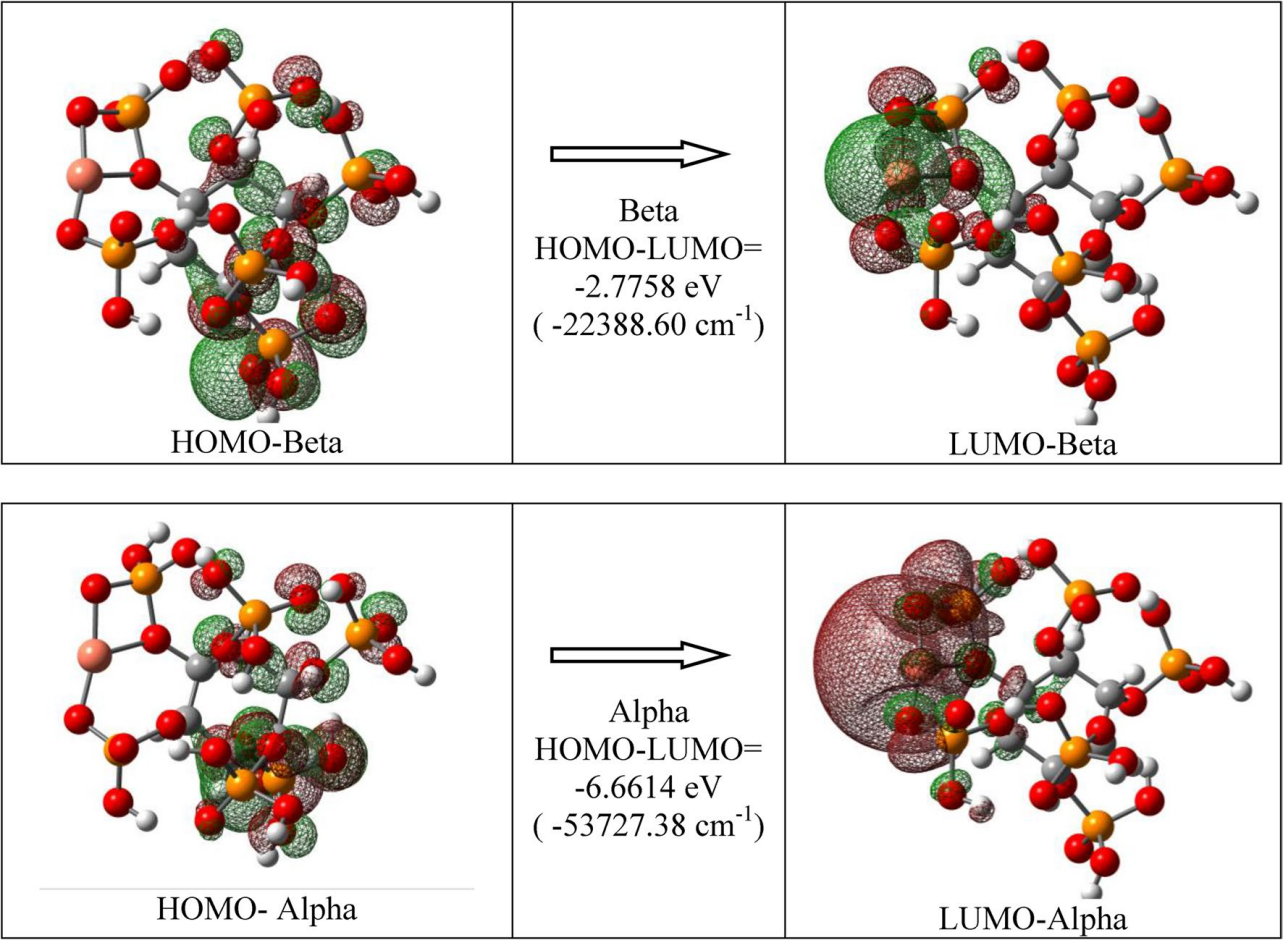
The view of the IP6 (left) and IP6Cu (right) molecules obtained from the geometry optimization in DFT calculations: (gray)—carbon, (red)—oxygen, (orange)—phosphorus, (light gray)—hydrogen atoms and (pink)—copper atom. In both structures the O–H···O hydrogen bonds are shown

In the DFT procedure, the geometry optimization of the IP6Cu complex was performed using the XRD data reported by Blank et al. [25] for phytic acid sodium salt. The comparison of these structural parameters with the ones calculated in the present work is shown in Table 1. A good agreement between these data proves that the B3LYP/6-31G(d,p) approach used in the DFT calculations is satisfactory. Figure 2 shows the geometry of the IP6 and IP6Cu molecules derived from the DFT optimisation.

**Table 1:** Comparison of the experimental structural data (bond lengths and angles) with calculated by us geometrical parameters

Structural parameters	Experimental data [25]	Calculated parameters
Bond lengths (Å)		
C–C	1.494–1.575	1.532–1.550
C–O_P_	1.400–1.470	1.430–1.458
O_p_–P	1.616–1.647	1.664–1.770
P=O	1.492–1.506	1.597–1.704
P–O_H_	1.506–1.527	1.577–1.618
Na–O or (Cu–O)	2.191–2.899	1.807–1.987
Angles (°)		
O–Cu–O	–	166.8
P–O–Cu	–	101.6–113.6
O–P–O	99.0–115.0	99.6–118.3
P–O–C	116.9–121.8	116.0–132.2
C–C–C	104.1–112.6	110.0–114.6
Hydrogen bonds D·····A (Å)		
Intramolecular O–H·····O	2.611–2.777	2.27–2.72
Intermolecular O–H·····O	2.813–3.052	–
O–H·····O angle (°)	150–176	139.3–167.4

It should be noted that the DFT calculations were performed for the isolated IP6 and IP6Cu molecules, whereas the XRD data [25] concern the system built of four molecules in the unit cell.

### Vibrational spectra

**Fig. 3:**
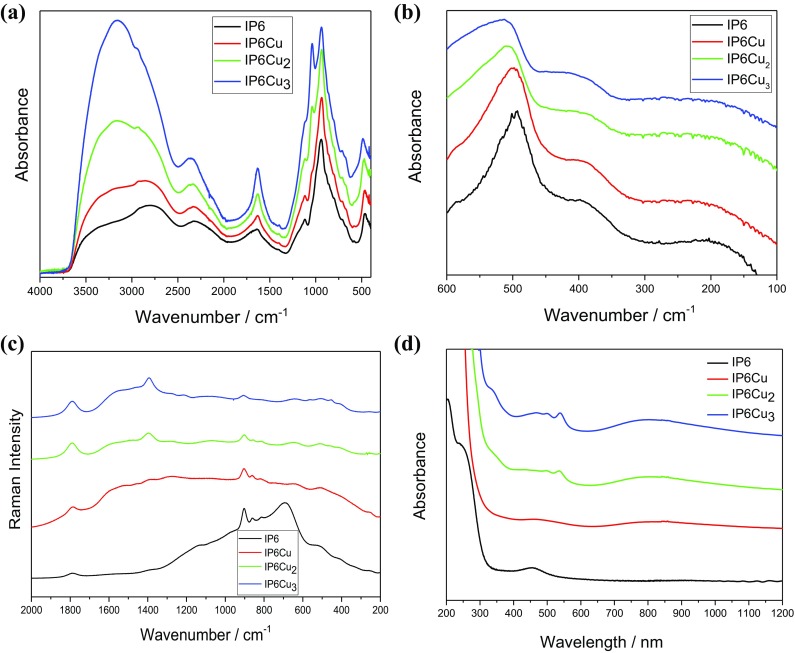
FT-MIR (**a**), FT-FIR (**b**), Raman (**c**) and electron absorption (**d**) spectra of the studied IP6 and its copper IP6Cu, IP6Cu_2_ and IP6Cu_3_ complexes

The IR spectra of the studied IP6, IP6Cu, IP6Cu_2_ and IP6Cu_3_ derivatives are shown in Fig. 3a, b in the MIR and FIR regions, respectively. Figure 3c shows the Raman spectra measured for all the compounds studied in this work.

The observed IR and Raman bands were assigned to the respective vibrations using these data. Their wavenumbers remain practically the same for all the studied compounds. They correspond to the following normal modes: *ν*(H_2_O) 3348 m,sh; *ν*(O–H···O) 3244 w,sh; 3158 s; *ν*(CH) 2944 sh, 2935 m; *ν*(A, B, C)—Fermi resonance: 2790 s,sh; 2341 m, 2137 sh; *δ*(HOH) 1770 sh, 1688 sh, 1629 m; *δ*(CH) 1396 w; *δ*(O–H···O) 1212 w,sh; *ν*(*φ*) + *δ*(CH) 1153 sh; *ν*(C–O) 1046 m,sh; *ν*_as_(PO_4_) 1116 m, 1039 sh, 937 vs; *ν*_s_(PO_4_) 902 sh; *γ*(CH) 889 sh; *γ*(OH) and *γ*(O–H···) 859 sh, *ν*(*φ*) ring breathing + *δ*(C–O–P) 802 sh; *γ*(OH) + *ν*(PO_4_) 707 m; 668 m; *γ*(*φ*) 632 sh, 614 w; *δ*(C–O–P) 580 w, 553 sh; *δ*_as_(PO_4_) 463 m, 454 m; *δ*_s_(PO_4_) 418 w; *ν*(CuO_6_) 150–280 w, vb. The complex band at 1000 cm^−1^, corresponding mainly to the *ν*_as_(PO_4_) and *ν*_s_(PO_4_) stretching vibrations, dominates the whole spectral band and practically has the same energy regardless of the growing amount of Cu^2+^ cation but its intensity strongly increases in this series. On the other hand, the maximum of the *δ*_as_(PO_4_) band shifts in the studied series from 480 cm^−1^ for IP6, 488 cm^−1^ for IP6Cu, 495 cm^−1^ for P6Cu_2_ to 510 cm^−1^ for IP6Cu_3_. It should be noted that the shape of all these bands slightly changes in this series, which means that the local *C*_s_ symmetry of the phosphate anion for IP6 changes to *C*_1_ for the complexes.

The Raman spectra presented in Fig. 3c contain bands similar to those seen in the IR spectra. They are dominated by vibrations of the hydroxyl groups engaged in the hydrogen bonds, phosphate groups and ester C–O–P linkage. The following vibrations are active in the Raman spectra: 1788–1791 and 1600 cm^−1^ − *δ*(H_2_O) and *ν*(O–H····O); 1393–1395 cm^−1^ − *δ*(CH); 1274–1278 and 1066–1069 cm^−1^ − *ν*(C–O) + *ν*_as_(PO_4_); 902–905 and 859–860 cm^−1^ − *ν*_s_(PO_4_), 810–815 cm^−1^ − *ν*(*ϕ*); 634–640 cm^−1^ − *γ*(OH) + *ν*(PO_4_); 503–506 cm^−1^ − *δ*(C–O–P) + *δ*(PO_4_); 454 and 401–403 cm^−1^ − *ν*(Cu–O) + *δ*_s_(PO_4_); 260–264 and 190–193 cm^−1^ − *ν*(Cu–O) + *ν*(O·····H); 190–193 and 143 cm^−1^*ν*(O·····H). The symmetric vibrations of the phosphate group are observed in the ranges 902–905 and 859–860 cm^−1^ as strong and narrow bands. The other bands are broad with medium to weak intensity. It should be noted that other weak and broad bands in the range 140–265 cm^−1^ correspond to the vibrations of the copper–oxygen polyhedron and fit well similar bands observed by Sakai et al. [26] for other metal–phytates in the range 100–500 cm^−1^.

Supplementary Figure S1 shows visualization of selected vibrational modes proposed for the studied IP6Cu complex. In Table S1, a comparison of the theoretical and experimental wavenumber for the IP6Cu is presented.

The recorded spectra are a good illustration of the structure described by the XRD data [25] and presented DFT calculations. They consist of a few clearly distinguishable vibrational bands corresponding to the phosphate units, C_6_H_6_ ring and different types of hydroxyl groups involved in different interactions. This is particularly visible when the intensity and shape of the very broad bands in the range 2000–3750 cm^−1^ are compared. For IP6, the component of this band ranging from 3150 to 3250 cm^−1^ exhibits a lower intensity than those of at 2790 and 2341 cm^−1^. The growing content of copper ions in the series IP6Cu, IP6Cu_2_, IP6Cu_3_ causes a gradual intensity increase of the band at 3158 cm^−1^. Having in mind, that each copper ion coordinates at least two water units, a gradual increase of the water amount accompanies this change.

The dependence of the intensity and shape of the main bands observed in the spectra of IP6 and its copper complexes in the ranges 2000–3750, 500–1250 and 300–600 cm^−1^ allows to propose the structure of the CuO_6_ coordination polyhedron. Cu^2+^ ion, substituting the hydrogen atom of the OH group, coordinates six oxygen atoms: two from this hydroxyl group, two oxygen atoms of the P=O groups and two oxygen atoms from the water units present in the structure of the resin material. The structure of the whole CuO_6_ polyhedron could be described as a strongly distorted octahedron. The calculated Cu–O distances range from 1.807 to 1.987 Å.

In the present work, we use quantum chemical DFT calculations in the analysis of the IR and Raman spectra measured in the 50–4000 cm^−1^ range for the IP6, IP6Cu, IP6Cu_2_ and IP6Cu_3_ complexes. Work assignment of the bands presented confirms in several details the results described in the earlier papers in which the vibrational spectra were measured and analyzed [26–30].

### Electron absorption spectra

Electron absorption spectra of the studied phytic acid and its copper complexes are shown in Fig. 3d.

The spectra of IP6 and its copper complexes reveal a strong absorption at 150–300 nm with a weak shoulder at 300 nm (33,330 cm^−1^) and clear, but significantly less intense, bands at 450 nm (22,200 cm^−1^), 550 nm (18,180 cm^−1^) and 760 nm (13,160 cm^−1^). The intensity of these spectral bands increases with the increasing copper amount. For all the studied materials, the third band is observed in the range 350–700 nm. The band in the range 150–300 nm corresponds to the charge-transfer transition between the phosphate anion and copper cation. The HOMO → LUMO transition was evaluated for IP6 and IP6Cu complexes using the same basis and functional as the one applied for vibrational levels. The energies of the electron transitions calculated in such a way are presented in Table 2, for the IP6Cu complex they agree with the positions observed in the spectra (Fig. 3d).

**Table 2:** Singlet excited states for IP6 and IP6Cu complex

State number	IP6		IP6Cu	
	Energy (nm)	Oscillator strength	Energy (nm)	Oscillator strength
1	177.15	0.0016	896.14	0.0005
2	175.25	0.0043	524.57	0.0020
3	171.21	0.0017	444.14	0.0061
4	170.39	0.0044	364.83	0.0121
5	165.44	0.0129	347.69	0.0090
6	164.80	0.0132	336.23	0.0140
7	164.23	0.0025	295.88	0.0046
8	163.67	0.0061	262.69	0.0028
9	162.83	0.0054	252.14	0.0027
10	161.47	0.0021	249.40	0.0023

The spectra associated with Cu^2+^ ion are *d*–*d* transitions which usually are analyzed in terms of the ligand field theory. The term of the free copper(II) (*d*^9^) is ^2^*D*. It splits into lower ^2^*E*_g_ and higher ^2^*T*_2g_ in octahedral crystal field. Jahn–Teller effect leads to tetragonal or rhombic distortion of the coordination polyhedron for which the ^2^*E*_g_ level splits into ^2^*A*_1_ and ^2^*B*_1_ but ^2^*T*_2g_ level into ^2^*E* and ^2^*B*_2_ levels, the ground state is ^2^*B*_1_. This structure is usually more energetically favored than that of regular octahedron. Further symmetry lowering from *D*_4h_ to, e.g., *C*_2v_ creates a new energy level sequence: *B*_1_(*x*^2^−*y*^2^), *A*_1_(*z*^2^), *A*_1_(*xy*), *A*_2_(*xz*) and *B*_2_(*yz*). Therefore, a complex spectral band is expected for the CuO_6_ distorted octahedron appearing in the copper phytate, similarly to the other Cu(III) compounds [31–37]. Indeed, the electron absorption spectra of the IP6Cu, IP6Cu_2_ and IP6Cu_3_ complexes shown in Fig. 3d exhibit the following *d–d* transitions: ^2^*B*_1g_ → ^2^*E*_g_ 22,200 cm^−1^ (450 nm) and 18,180 cm^−1^ (550 nm); ^2^*B*_1g_ → ^2^*B*_2g_ 13,160 cm^−1^ (760 nm); ^2^*B*_2_ → ^2^*E* 5260 cm^−1^ (1900 nm) and 5050 cm^−1^ (1980 nm). Such a spectroscopic behavior proves that the CuO_6_ unit appears in the studied complexes in the form of elongated along the *z*-axis octahedron and the ground state of Cu^2+^ ions is the *x*^2^–*y*^2^ orbital (^2^*B*_1g_ state). The clear splitting of the ^2^*E*_g_ level seen in the spectra results from the strong structural distortion of the coordination polyhedron. The appearance and splitting of the ^2^*B*_2_ → ^2^*E* transition is particularly expressive. As the copper amount increases in the studied complexes, the growing intensity and a slight shift of the bands are observed and their splitting becomes more distinct.

### EPR spectra measurements: magnetic properties

**Fig. 4:**
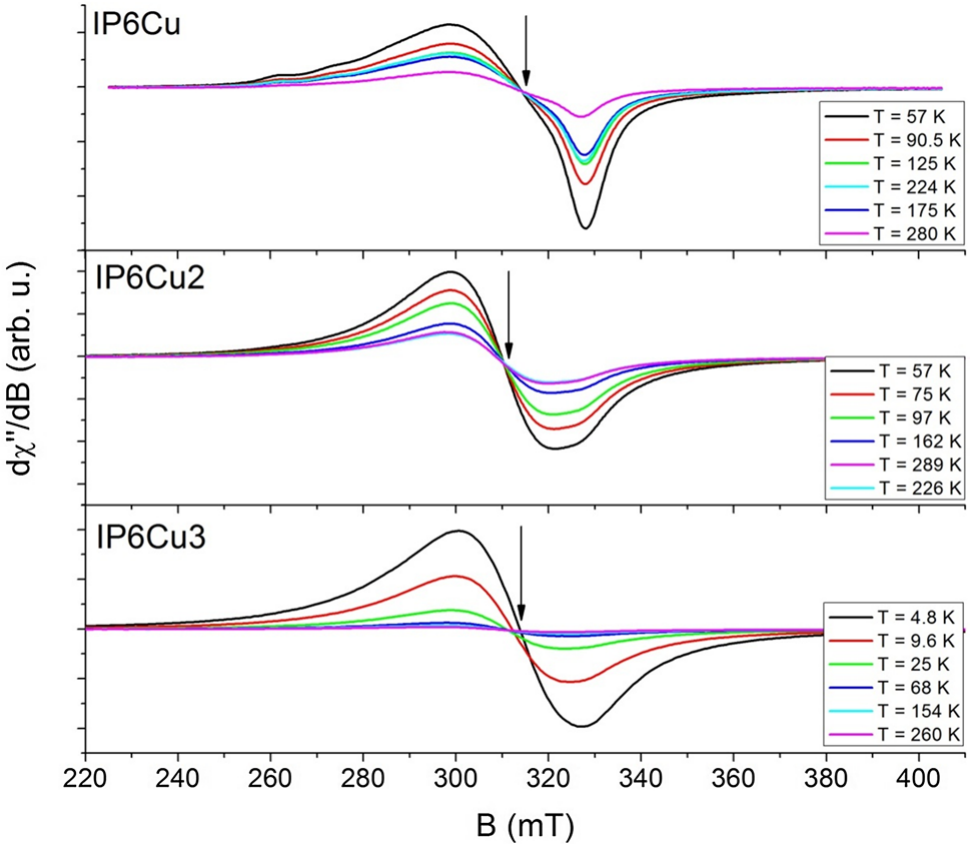
EPR spectra of the IP6Cu (upper panel), IP6Cu_2_ (middle panel), IP6Cu_3_ (bottom panel) complexes for several temperatures

**Fig. 5:**
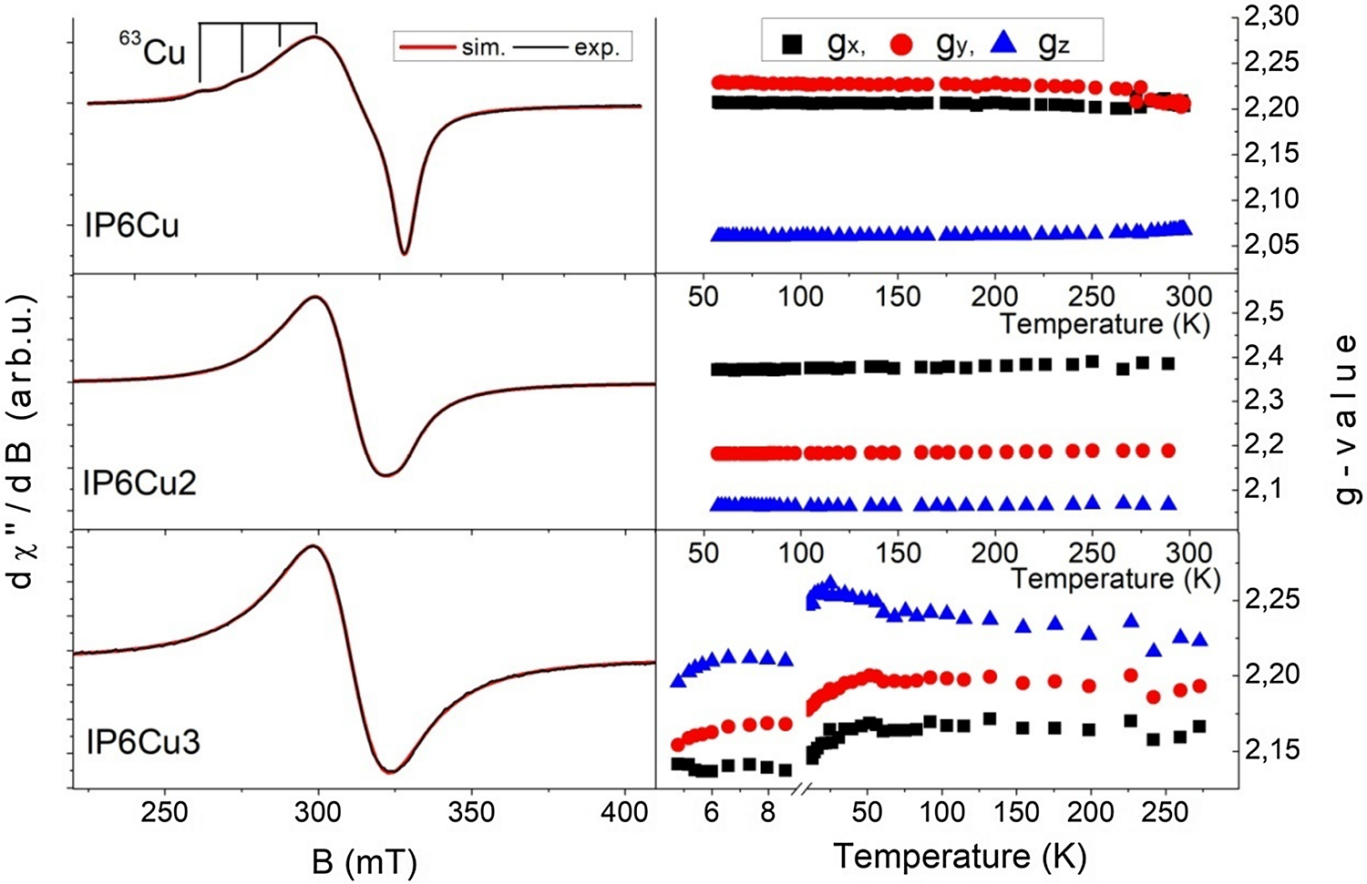
Experimental EPR spectra measured at ~ 60 K and a fitting curve obtained from SIMPOW6 program for Cu complexes (left panel). Spectroscopic *g* factor (right panel) vs. temperature for the same complexes

The EPR spectra of the IP6Cu*x* (*x* = 1, 2, 3) complexes recorded at several temperature values are presented in Fig. 4. The EPR signal is observed in the range 220–400 mT and it originates from copper ions with a spin of *S* = ½. It is an asymmetric one and it is visible in the whole experimental temperature range. Depending on the value of *g* factor parameters, the ground state of copper ion could be *d*_*z*_^2^ or *d*_*x*_^2^_− *y*_^2^. Copper has two magnetic isotopes ^63^Cu (abundance ~ 69%) and ^65^Cu (abundance ~ 31%) with a nucleus spin *I* = 3/2. As one can see in Fig. 4, weak hyperfine lines derived from the ^63^Cu isotope are only visible in the EPR spectrum of the IP6Cu complexes (Fig. 5).

Spin Hamiltonian parameters of the IP6Cu, IP6Cu_2_ and IP6Cu_3_ complexes were calculated in the present work applying the SIMPOW6 program [23] to fit the experimental EPR spectra. The results of the fittings are shown in Fig. 5 (left panel) and they are summarized in Table 3. Three different *g* values were obtained. The large anisotropies of the *g* tensor permit a qualitative establishment of the appropriate ground state wavefunction. When the factor given by *R* = (*g*_1_−*g*_2_)/(*g*_3_−*g*_1_), where (*g*_3_ > *g*_1_ > *g*_2_) is greater than unity, a predominantly *d*_*z*_^2^ ground state exists, while for *R* < 1, a predominant *d*_*x*_^2^_– *y*_^2^ state would be expected [38, 39]. The observed values for the Cu^2+^ sites suggest *d*_*z*_^2^ ground state for IP6Cu, and, *d*_*x*_^2^_– *y*_^2^ for the IP6Cu_2_ and IP6Cu_3_ complexes. The local symmetry of copper ions seems to be rhombic like.

**Table 3:** Spin Hamiltonian parameters at a temperature of *T* ~ 60 K for copper complexes

*g* value	Complexes		
	IP6Cu	IP6Cu_2_	IP6Cu_3_
*g*_*x*_	2.23 (2)	2.37 (2)	2.24 (2)
*g*_*y*_	2.21 (1)	2.18 (1)	2.20 (1)
*g*_*z*_	2.06 (2)	2.07 (2)	2.17 (2)

The *g* values are given in the standard notation applied for powder compounds—*g*_*x*_ > g_*y*_ > g_*z*_, where *x*, *y*, *z* laboratory axis system

For the IP6Cu complex, a hyperfine structure with the value of hyperfine constant *A* = 12 mT was observed. It is a frequent case when the hyperfine splitting is resolved in a powder EPR spectrum of a Cu(II) complex suggesting a higher isolation of Cu(II) centers. The values of the *g* factors are constant almost in the whole measurement temperature range *T* = 57–273 K, as it can be seen in Fig. 5 (right panel). The EPR line width remains constant up to *T* = 224 K, then it slightly decreases in the case of Δ*B*_*x*_ and Δ*B*_*y*_ values, while slightly increases in the case of Δ*B*_*z*_ value with increasing temperature. Significant changes in *g* factors and Δ*B* values are observed at about *T* = 273 K. The changes may be due to the presence of water molecules in the IP6Cu complex, being weakly bound to a ligand.

In the case of the IP6Cu_2_ complex, for which EPR spectra were recorded in the same temperature range as for the IP6Cu complex, no significant changes in *g* and Δ*B* parameters were observed (Fig. 5, right panel).

The IP6Cu_3_ complex was investigated in the temperature range 4–300 K. In the temperature range 4–9 K, the *g* values are increasing while the EPR line-width values are decreasing with increasing temperature. At the temperature *T* = 9 K, there is a sudden change in the *g* value and in the behavior of the observed EPR line width. The changes may suggest a change in the magnetic phase or a structural change of the complex. The values remain constant at the observed temperature *T* = 55 K.

**Fig. 6:**
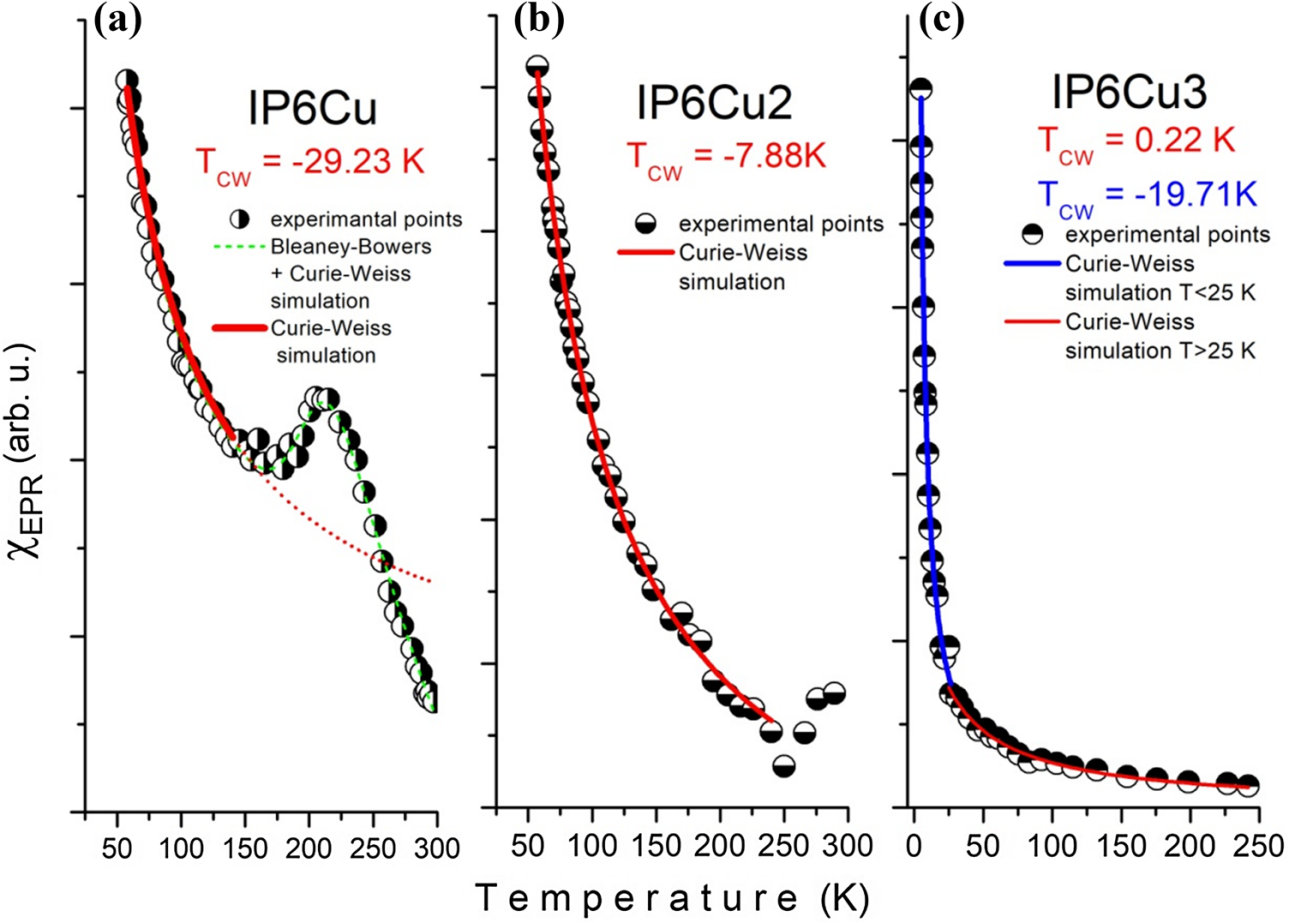
Temperature dependences of EPR integrated intensity calculated for **a** IP6Cu complex (left panel), **b** IP6Cu_2_ complex (middle panel) and **c** IP6Cu_3_ complex (right panel). Solid line is the best fit of the modified Bleaney–Bowers and Curie–Weiss equations to the experimental curve

The temperature dependence of the total intensity of the EPR signal is shown in Fig. 6a–c for the studied copper complexes. The total intensity is proportional to the EPR magnetic susceptibility, *χ*_EPR_. It can be fitted by the Curie–Weiss law, giving *T*_CW_, Curie–Weiss temperature (sometimes called Weiss constant or Weiss temperature). As one can see in Fig. 6a (left panel), the EPR magnetic susceptibility of IP6Cu is a complex one. In the temperature range 57 K < *T* < 140 K, the intensity of the EPR signal decreases in accordance with the Curie–Weiss law, then increases to *T* ~ 210 K, and then decreases again to the room temperature. This is a characteristic behavior usually observed for copper dimers with a spin of *S* = 1. However, the EPR signal attributed to a spin *S* = 1 is not observed in the EPR spectrum (see Fig. 5). It is typical for isolated copper ions. We fitted the dependence of the EPR intensity to the modified Bleaney–Bowers + Curie–Weiss equation, obtaining the interaction constant, *J*, with an unreal large value. Therefore, we think that the increase in the EPR intensity can be caused rather by a thermal filling of the excited levels, for which the energy between the ground state is *Δ* ~ 146 cm^−1^. Temperature investigations show strong antiferromagnetic interactions observed for the IP6Cu complex, somewhat weaker antiferromagnetic interactions for IP6Cu_2_, and a lack of magnetic interaction for the IP6Cu_3_ complex. The IP6Cu_3_ complex was also studied at helium temperature. The results of the total intensity vs. temperature show strong antiferromagnetic interactions in the low temperature range (*T* < 25 K). A change in the total intensity correlates with the decrease in *g* value (*g*_*x*_, *g*_*y*_, *g*_*z*_), which suggests a decrease in the distance between paramagnetic ions. The comparison of the spin Hamiltonian parameters for the studied complexes is presented in Table 3.

All the studied complexes reveal a local rhombic symmetry of copper ions. Dominant interactions are of an antiferromagnetic nature, depending on the amount of paramagnetic ions. These results agree with those reported in other works concerning Cu^2+^ ions in oxygen environment [34, 40–42]. On the basis of the structure of a similar compound, myoinositol hexaphosphate dodecasodium salt, a probable origin of the antiferromagnetic coupling could be proposed. According to the XRD data, this salt crystallizes in the Cc monoclinic structure with the cell parameters *a* = 23.091, *b* = 12.203, *c* = 22.894 Å, *β* = 108.300° and *Z* = 4 [25]. In the solid state, the phytate ring adopts the chair configuration, which is so stable that it also exists in the solution. The metal ions are coordinated to the oxygen atoms of the phosphate units that appear at the equatorial or vertical positions of the IP6 ring. Therefore, the spins of these ions form two ordered sets differing in the direction of the magnetic moment. When Cu^2+^ ions built such sets they may interact with each other by mediation of the phosphate units playing the role of the bridges joining the magnetic ions. Such a situation is particularly effective for the IP6Cu molecule for which the relation IP6:Cu is 1:1. For a greater amount of the magnetic ions, the compensation in magnetic moments can occur leading to weaker antiferromagnetic interactions for the IP6Cu_2_, and lack of magnetic interaction for the IP6Cu_3_ complex.

## Conclusion

The following conclusions can be drawn from the spectroscopic and magnetic studies of the IP6Cu, IP6Cu_2_ and IP6Cu_3_ complexes:

- The IR and Raman spectra are dominated by the vibrations of the phosphate units as well as both intermolecular and intramolecular O–H···O interactions between the O–H and P=O bonds of the phosphate ions and water units present in the space structure,
- The local structure of the phosphate unit is *C*_s_ in non-substituted H_2_PO_4_^−^ phosphate. The substitution of Cu^+^ ions leads to lowering of the symmetry to C_1._
- The *ν*(Cu–O) vibrations are observed in the 200–510 cm^−1^ range and these motions are strongly coupled with vibrations of the phosphate groups,
- The electron absorption spectra of the studied materials are characterized by a specific triplet of the bands observed at about 450, 550, 760 nm and an additional weak band at 1900 nm. Their intensity strongly depends on the amount of copper content. They correspond to the *d*–*d* transitions and their doublet character results from a strong distortion of the CuO_6_ polyhedron,
- The copper–oxygen polyhedron is built of two oxygen atoms originated from hydroxyl groups, two oxygen atoms of the P=O groups and two oxygen atoms of the water molecules present in the structure of the resin material. The calculated Cu–O distances range from 1.807 to 1.987 Å.
- The rhombic distortion of the CuO_6_ polyhedron has been confirmed by the results of the EPR studies in which the Hamiltonian parameters were characterized. The antiferromagnetic interactions bind Cu^2+^ ions in the structure of the studied complexes.
- The copper amount influences both spectroscopic and magnetic properties of the studied complexes visible in the change of some band intensities and their splitting.

## Electronic supplementary material

Below is the link to the electronic supplementary material.

Supplementary material 1 (PDF 327 kb)

Supplementary material 2 (PDF 393 kb)

## References

[1] Ronen E (2007) Micro-elements in agriculture. The importance of micro-elements. In: Practical hydroponics & greenhouses. Casper Publications Pty Ltd, Narrabeen, pp 39–48

[2] Zając A, Dymińska L, Lorenc J (2018). Syntheses, spectroscopic properties and molecular structure of silver phytate complexes—IR, UV–VIS studies and DFT calculations. J Mol Struct.

[3] He Z, Zhong J, Cheng HN (2013). Conformational change of metal phytates: s olid state 1D 13 C and 2D 1 H- 13 C NMR spectroscopic investigations. J Food Agric Environ.

[4] Cosgrove DJ, Irving GCJ (1980). Inositol phosphates: their chemistry, biochemistry, and physiology.

[5] Choi YM, Suh HJ, Kim JM (2001). Purification and properties of extracellular phytase from *Bacillus* sp. KHU-10. J Protein Chem.

[6] Kim Y-O, Kim H-K, Bae K-S (1998). Purification and properties of a thermostable phytase from *Bacillus* sp. DS11. Enzyme Microb Technol.

[7] Kerovuo J, Lauraeus M, Nurminen P (1998). Isolation, characterization, molecular gene cloning, and sequencing of a novel phytase from *Bacillus subtilis*. Appl Environ Microbiol.

[8] George TS, Quiquampoix H, Simpson RJ, Richardson AE, Richardson AE, Mullaney EJ (2007). Interactions between phytases and soil constituents: implications for the hydrolysis of inositol phosphates. Turner BL.

[9] Champagne ET (1988). Effects of pH on mineral-phytate, protein-mineral-phytate, and mineral-fiber interactions. Possible consequences of atrophic gastritis on mineral bioavailability from high-fiber foods. J Am Coll Nutr.

[10] Morel FÃMM, Hering JG (1993). Principles and applications of aquatic chemistry.

[11] Stumm W, Morgan JJ (1996). Aquatic chemistry: chemical equilibria and rates in natural waters.

[12] Heighton L, Schmidt WF, Siefert RL (2008). Kinetic and equilibrium constants of phytic acid and ferric and ferrous phytate derived from nuclear magnetic resonance spectroscopy. J Agric Food Chem.

[13] O’Dell BL, De Boland A (1976). Complexation of phytate with proteins and cations in corn germ and oil seed meals. J Agric Food Chem.

[14] Greiner R, Ravindran V, Bryden WL, Kornegay E (1995). Phytates: occurrence, bioavailability and implications in poultry nutrition. Poult Avian Biol Rev.

[15] Angel R, Tamim NM, Applegate TJ (2002). Phytic Acid chemistry: influence on phytin-phosphorus availability and phytase efficacy. J Appl Poult Res.

[16] Frisch MJ, Trucks GW, Schlegel HB et al (2003) Gaussian 03, Revision A.1

[17] Becke AD (1996). Density-functional thermochemistry. IV. A new dynamical correlation functional and implications for exact-exchange mixing. J Chem Phys.

[18] Lee C, Yang W, Parr RG (1988). Development of the Colle–Salvetti correlation-energy formula into a functional of the electron density. Phys Rev B.

[19] Parr RG, Yang W (1989). Density-functional theory of atoms and molecules.

[20] McLean AD, Chandler GS (1980). Contracted Gaussian basis sets for molecular calculations. I. Second row atoms, Z = 11–18. J Chem Phys.

[21] Krishnan R, Binkley JS, Seeger R, Pople JA (1980). Self-consistent molecular orbital methods. XX. A basis set for correlated wave functions. J Chem Phys.

[22] Zhurko GA, Zhurko DA Chemcraft. https://www.chemcraftprog.com

[23] Nilges MJ (1979). Thesis in chemistry.

[24] Mombourquette MJ, Weil JA, McGavi DG (1999). EPR-NMR user’s manual.

[25] Blank GE, Pletcher J, Sax M (1975). Hemoglobin cofactors. I. The crystal structure of myoinositol hexaphosphate dodecasodium salt octatriacontahydrate. Acta Crystallogr Sect B Struct Crystallogr Cryst Chem.

[26] Sakai H, Ikemoto Y, Kinoshita T (2017). Fourier-transform spectra of metal salts of phytic acid in the mid- to far-infrared spectral range. Vib Spectrosc.

[27] Heighton LP, Zimmerman M, Rice CP (2012). Quantification of inositol hexa-kis phosphate in environmental samples. Open J Soil Sci.

[28] Kassama LS, Sanusi TO (2016). Characterization of phytic acid in tempered canned red kidney beans (*Phaseolus vulgaris*) using Raman spectroscopy. GSTF J Agric Eng.

[29] He Z, Honeycutt CW, Zhang T, Bertsch PM (2006). Preparation and FT–IR characterization of metal phytate compounds. J Environ Qual.

[30] He Z, Honeycutt CW, Xing B (2007). Solid-state fourier transform infrared and 31p nuclear magnetic resonance spectral features of phosphate compounds. Soil Sci.

[31] Sever MJ, Wilker JJ (2004). Visible absorption spectra of metal–catecholate and metal–tironate complexes. Dalton Trans.

[32] Pascual JL, Savoini B, González R (2004). Electronic absorption spectra of Cu^2+^ in MgO: Ab initio theory and experiment. Phys Rev B.

[33] Dhanuskodi S, Angeli Mary PA, Sambasiva Rao P (2005). Single crystal EPR and optical absorption studies of Cu^2+^ ion in l-arginine sulphophosphate monohydrate—a nonlinear optical crystal. Spectrochim Acta Part A Mol Biomol Spectrosc.

[34] Rao NS, Bale S, Purnima M (2005). Optical absorption and electron spin resonance studies of Cu^2+^ in Li_2_O–Na_2_O–B_2_O_3_–As_2_O_3_ glasses. Bull Mater Sci.

[35] Lakshminarayana G, Buddhudu S (2005). Spectral analysis of Cu^2+^: B_2_O_3_–ZnO–PbO glasses. Spectrochim Acta Part A Mol Biomol Spectrosc.

[36] Barskaya IY, Veber SL, Suturina EA (2017). Spin-state-correlated optical properties of copper(II)–nitroxide based molecular magnets. Dalton Trans.

[37] Morsi RMM, Ibrahim S, Morsi MM (2017). Preparation and characterization of materials in the system xCuO–(50-x) CdO–50B_2_O_3_. Ceram Int.

[38] Dudley RJ, Hathaway BJ (1970). Single-crystal electronic and electron spin resonance spectra of dichlorobis-(2-methylpyridine)copper(II). J Chem Soc A Inorganic Phys Theor.

[39] Narasimhulu KV, Sunandana CS, Rao JL (2000). Electron paramagnetic resonance studies of Cu^2+^ ions in KZnClSO_4_·3H_2_O: an observation of Jahn–Teller distortion. J Phys Chem Solids.

[40] Siegel I, Jones EP (1972). Electronic bonding of Cu^2+^ in amorphous and crystalline TeO_2_: EPR and optical spectra. J Chem Phys.

[41] Noh TH, Le Shim E (2017). Study of CuO content on physical and structural properties of Li_2_O–B_2_O_3_–CuO glasses using electron paramagnetic resonance. J Non Cryst Solids.

[42] Jacobsen T, Slotfeldt-Ellingsen D (1983). Phytic acid and metal availability: a study of Ca and Cu binding. Cereal Chem.

