# Supplementary material for: Spectroscopic properties and molecular structure of copper phytate complexes: IR, Raman, UV–Vis, EPR studies and DFT calculations
Source: J Biol Inorg Chem. 2018 Oct 24;24(1):11–20. doi: 10.1007/s00775-018-1622-0 (PMC6394811; doi:10.1007/s00775-018-1622-0)
Supplement: Supplementary file 1 — Supplementary material 1 (PDF 327 kb) [file 775_2018_1622_MOESM1_ESM.pdf]

Table S1. The comparison of the theoretical and experimental wavenumber for the IP6Cu.

**Calc. wavenumb er**	**Relative intensity%***	**IR exp.**	**RS exp.**	**Assignment**
3743	5	3348sh		$\nu(H_2O)$
3741	4			
3732	6			
3710	8			
3392	16	3244w 3158sh		ν(O-H···O) intermolecular
3224	28			
3119	44			
3119	4			
2962	47	2944sh 2935m		ν(CH)
		2790s		ν(A,B,C) – Fermi resonance
2399	46	2341m		
2346	100	2137sh		
		1770 sh 1688 sh 1629m	1788-1791, 1600	$\delta(H_2O)$
1466	7		1463	ν(OH···O)
1315	1	1396w	1393-1395	δ(CH)
1312	1			
			1274-1278	+ $\nu_{as}(PO_4)$
1300	4	1212w		δ(OH···O)
1292	3			
1259	6			
1230	5			
1192	7	1153sh		$\nu(\varphi) + \delta$(CH)
1151	4			
1137	3			
1122	5	1116m		$\nu_{as}(PO_4)$
1117	6			
1106	4			
1102	6	1046m	1066-1069	ν(C-O) + $\nu_{as}(PO_4)$
1096	28			
1075	9			
1057	5	1039sh 937vs		$\nu_{as}(PO_4)$
1052	1			
1045	18			
1028	4			
1023	7			
1018	4	902sh	902-905	$\nu_s(PO_4)$
1015	20			
1010	2			
1005	5			
1000	1			

993	2			
986	4	889sh		γ(CH)
941	3			
931	4			
908	12			
903	3			
880	3			
872	9	859sh	859-860	γ(OH) + γ(O-H···) + $\nu_s(PO_4)$
864	3			
842	12			
836	8			
816	7			
810	3	802sh	810-815	ν(φ) ring breathing + δ(C-O-P)
799	14			
793	4			
786	2			
779	2			
762	4	707m<br>668m	634-640	γ(OH) +$\nu(PO_4)$
749	2			
735	8			
729	8			
728	4			
708	4	632sh<br>614w		γ(φ)
671	2			
655	4			
632	2	580w<br>553sh		δ(C-O-P)
629	5			
608	1			
537	1	488m<br>454m	503-506	$\delta_{as}(PO_4)$
525	1			
498	2			
471	1			
459	2			
441	2	418w	401-403	$\delta_s(PO_4)$ +ν(CuO)
421	3			
418	3			
409	2			
402	1			
390	2			
383	4			
381	2			
368	1			
360	3		260-264	ν(CuO) + ν(O···H)
337	6			
318	4			
284	1			
278	1			
275	1			

248	1			
232	4			
197	1			
195	2			
186	1			
180	1	150	143	ν(O···H) + ν(CuO_6)
106	1			

abbreviations used: s, strong; m, medium; w, weak; v, very; sh, shoulder; ν, stretching (s, symmetric; as, asymmetric); δ, scissoring; and φ, inositol ring;

* In the comparison of the theoretical and experimental wavenumber for the IP6Cu modes having intensity greater than 1%.
