# Supplementary material for: Spectroscopic properties and molecular structure of copper phytate complexes: IR, Raman, UV–Vis, EPR studies and DFT calculations
Source: J Biol Inorg Chem. 2018 Oct 24;24(1):11–20. doi: 10.1007/s00775-018-1622-0 (PMC6394811; doi:10.1007/s00775-018-1622-0)
Supplement: Supplementary file 2 — Supplementary material 2 (PDF 393 kb) [file 775_2018_1622_MOESM2_ESM.pdf]

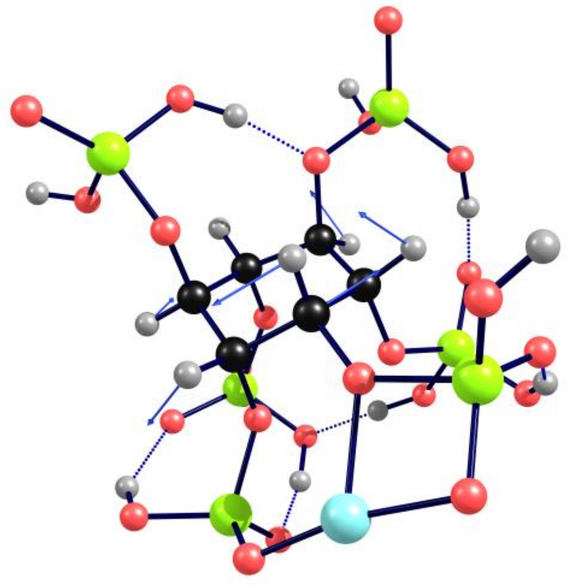

(a) 1393-1395 cm^{-1} (1395w cm^{-1})* - δ(CH)

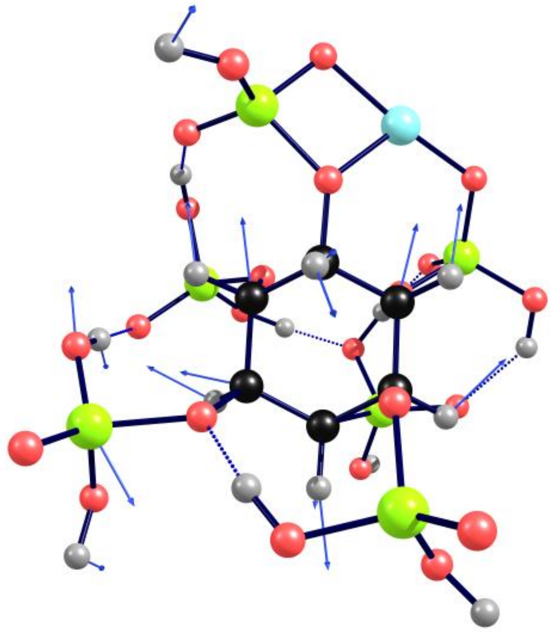

(b) 810-815 cm^{-1} (802sh cm^{-1})* - ν(φ)

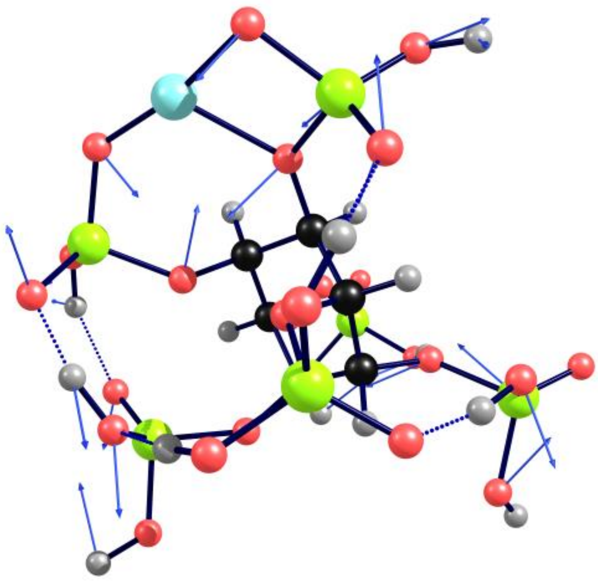

(c) 503-506 cm^{-1} (488m cm^{-1}; 454m cm^{-1})* - δ(C-O-P) + δ(PO_4)

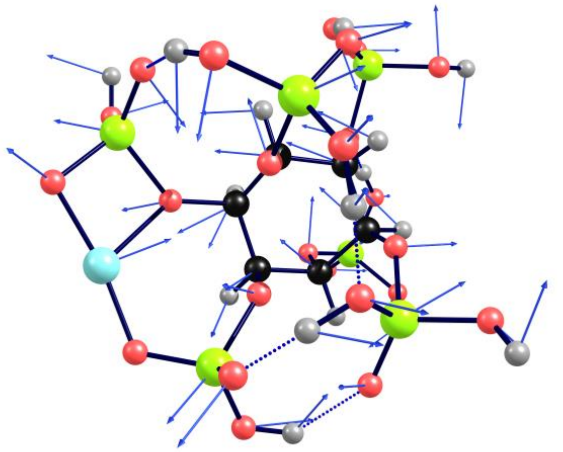

(d) 190-193 cm^{-1} (150w cm^{-1})* - ν(Cu-O) + ν(O···H)

Fig. S1. The visualisation of selected vibrational modes (created with ChemCraft).

* The observed wavenumbers are given in parentheses.

Abbreviations used: s, strong; m, medium; w, weak; v, very; sh, shoulder; ν, stretching (s, symmetric; as, asymmetric); δ, scissoring; and φ, inositol ring.
